# PYTHIA: Deep Learning Approach for Local Protein Conformation Prediction

**DOI:** 10.3390/ijms22168831

**Published:** 2021-08-17

**Authors:** Gabriel Cretin, Tatiana Galochkina, Alexandre G. de Brevern, Jean-Christophe Gelly

**Affiliations:** 1 Biologie Intégrée du Globule Rouge, Université de Paris, UMR_S1134, BIGR, INSERM, 75015 Paris, France; gabriel.cretin@u-paris.fr (G.C.); tatiana.galochkina@u-paris.fr (T.G.); alexandre.debrevern@u-paris.fr (A.G.d.B.); 2 Laboratoire d’Excellence GR-Ex, 75015 Paris, France

**Keywords:** protein structure, protein blocks, prediction, deep learning

## Abstract

Protein Blocks (PBs) are a widely used structural alphabet describing local protein backbone conformation in terms of 16 possible conformational states, adopted by five consecutive amino acids. The representation of complex protein 3D structures as 1D PB sequences was previously successfully applied to protein structure alignment and protein structure prediction. In the current study, we present a new model, PYTHIA (**p**redicting an**y** conforma**t**ion at **hi**gh **a**ccuracy), for the prediction of the protein local conformations in terms of PBs directly from the amino acid sequence. PYTHIA is based on a deep residual inception-inside-inception neural network with convolutional block attention modules, predicting 1 of 16 PB classes from evolutionary information combined to physicochemical properties of individual amino acids. PYTHIA clearly outperforms the LOCUSTRA reference method for all PB classes and demonstrates great performance for PB prediction on particularly challenging proteins from the CASP14 free modelling category.

## 1. Introduction

Protein structure can be described at different levels of granulometry. Classically, local protein organization at the residue level is described in terms of secondary structures: α-helices and β-strands. The assignment of regular secondary structures is based on the pattern of hydrogen bonds between the amino hydrogen and carboxyl oxygen atoms in the protein backbone and represents nearly fifty percent of protein residues on average. All the unassigned protein regions are classified as coils. While such a description provides essential information on protein structural local conformation, it lacks precision. A more complete secondary structure classification was implemented by the Define Secondary Structure of Proteins algorithm (DSSP) [1] by introducing classes such as turn, pi-helix, and bridge. Nevertheless, the suggested classifications remain rather limited and do not detect subtle changes in the local geometry of protein structures. In particular, coils represent a very important ratio of residues in the Protein Data Bank (PDB, [2]) and their comparison requires information in addition to the simple secondary structure assignment. Therefore, protein structural alphabets were developed in order to describe the diversity of local protein backbone conformations in more detail. One of the most widely used structural alphabets is called Protein Blocks (PBs) [3,4]. PBs correspond to 16 structural conformational states, which can be adopted by five consecutive amino acids. The encoding of complex protein structures (3D) in a PB sequence (1D) has already been successfully applied to protein structure alignment, protein structure prediction [5,6], and to the analysis of disordered protein ensembles [7].

The application of structural alphabets to problems of protein structure requires efficient tools for local conformation prediction directly from an amino acid sequence. Indeed, structural alphabets give users the ability to investigate and analyze protein properties such as protein dynamic and flexibility. Thus, Protein Blocks have been widely used to predict protein flexibility [8,9], backbone deformability [10,11], allosteric effects [12], protein disorders [7], and molecular dynamics [12,13,14].

Historically, the first PB prediction methods were based on simple statistical approaches, providing the prediction rates of 35% [3] to 48% [15]. These approaches did not consider evolutionary information. Then, two types of approaches using evolutionary data had emerged: tools for specific problems and more general machine learning-based (ML) methods. Among the methods developed for specific cases, we can mention the pinning approach [16]. This method is based on Bayesian approaches and predicts long protein structural fragments by gradual extension of the hits of five consecutive PBs (called structural words) in the database using an algorithm similar to PSI-BLAST [17]. More recently, the knowledge-based PB-kPRED approach was developed [18]. It can only be used in the context of strong local similarity, i.e., if the database contains no fragments that share a high sequence of similarity with the query, no prediction is possible. These results had been used a few years ago for the development of fold recognition approaches but with biases (a) in the management of missing regions for learning and (b) strong imbalances in the predicted PBs [19].

The most simple ML-based approach was proposed by Dong and collaborators [20]. It performs predictions using the sequence alone but coupled with external methods for the prediction of secondary structures and solvent accessibility producing a prediction rate of 45%. The authors base their analysis on the comparison with conventional secondary structure predictions and specific examples. SVM-PB-Pred uses sequence profiles (PSSM) and rather old approaches for the prediction of secondary structures from NPS@ and GOR4 methods [21] combined with a support vector machine (SVM) predictor, which provides minor improvement of the prediction accuracy compared to the Bayesian approach [15] and again with an imbalance between the predicted PBs.

LOCUSTRA [22] and svmPRATT [23] are based on similar methodologies: they both use SVM classifiers and evolutionary information. svmPRATT is a general-purpose tool that attributes discrete labels to protein residues using an SVM framework. It uses local sequence information (provided by the user) around each residue to build classification models. For PB prediction, svmPRAT-P uses the 16 one-versus-all models to predict the likelihood of a residue to be a member of each of the 16 PB classes and assigns the class with the highest likelihood value. LOCUSTRA is, in turn, based on two-layer SVMs. SVM layers were trained to predict a PB class for a sequence position encoded by 15 amino acids sliding windows with each residue described by a PSSM profile from PSI-BLAST [17] alignment and a one-hot encoding vector of a length equal to 21 (20 amino acids + 1 extremity flag) per position. Despite similarity between the approaches, LOCUSTRA predictions appeared to be more robust in the independent benchmarks with the Q16 average equal to 60.8% (while that of svmPRATT falls to 55%). Therefore, to the best of our knowledge, LOCUSTRA remains the reference method for Protein Blocks prediction from the amino acid sequence despite being published more than 10 years ago.

In November 2020, Google DeepMind′s team performed best at the CASP 14 biennial competition for protein structure prediction, obtaining outstanding results using the AlphaFold2 program. Despite very good results, AlphaFold is less performant on some CASP 14 targets (e.g., flexible protein regions and proteins that are components of multi-protein complexes), suggesting that there are some particular protein families that require further investigation [24,25]. Furthermore, in their recent paper of the structure prediction of the human proteome using AlphaFold [26], the DeepMind team stated that 35.7% of the residues predicted were of high accuracy (pLDDT > 90), while 58.0% of the total residues were predicted confidently (pLDDT > 70), leaving room for much improvement. From a methodological perspective, AlphaFold and other powerful 3D structure prediction methods such as RoseTTAFold [27] both require an important number of homologous sequences for their multiple sequence alignments (MSA), which makes the methods less performant for orphan proteins [28] and can also make it difficult to capture the impact of small sequence changes on the protein structure. Finally, AlphaFold2 requires a substantial amount of resources and computational time, which can make it not suitable for high throughput experiments such as those performed for computational protein design and variant analysis. Structural alphabet prediction methods, on the contrary, are much easier to install and requires lighter resources. Structural alphabet prediction can be also further used for the development of more sophisticated tools for the prediction of properties than protein local structure, such as the ones exposed in the first part of the introduction.

In the current study, we developed a new deep learning model for the prediction of the protein local conformations in terms of PBs directly from the amino acid sequence called PYTHIA (**p**redicting an**y** conforma**t**ion at **hi**gh **a**ccuracy). Our method outperforms the state-of-the-art tool LOCUSTRA for all of the 16 PB classes during cross-validation on the non-redundant dataset as well as for an independent test set of CASP14 free modelling targets. PYTHIA demonstrates particularly impressive results for the rarest types of local structures, therefore successfully recognizing the most subtle variations in backbone conformation. It provides the opportunity to efficiently explore the changes in the protein local conformation in response to the amino acid sequence variations, thus offering a number of important biological applications.

## 2. Results

PYTHIA performs prediction using a deep neural network trained on a non-redundant data set of protein structures (see Section 4.1). In order to keep maximum information contained in protein sequences, we combined physicochemical properties of individual amino acids with the evolutionary information extracted from the multiple sequence alignments of protein homologs (see Section 4.2).

**Figure 1:**
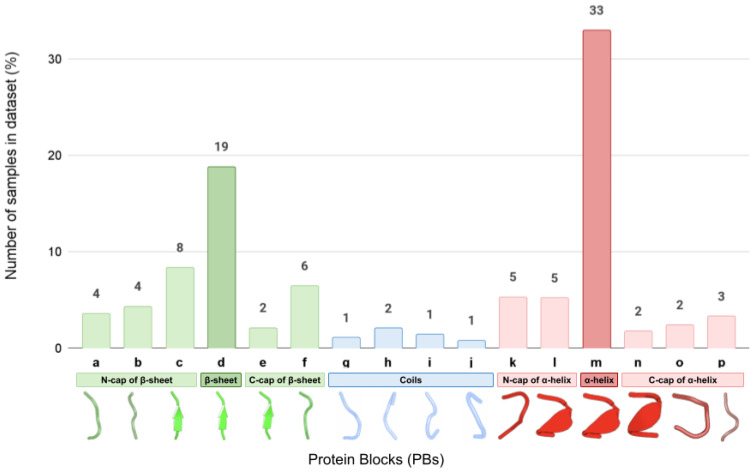
Number of samples (%) of each PB class in the full dataset.

In our study, we have paid particular attention to the presentation of the most rare PB structural classes. Indeed, the classes corresponding to the stable portions of the secondary structure motifs such as α-helices (PB ‘m’) and β-sheets (PB ‘d’) are overrepresented in our dataset and together constitute over 50% of all fragments (Figure 1). At the same time, PBs corresponding to helix and sheet caps (PBs ‘k’, ‘l’, ‘n’, ‘o’, and ‘p’, and ‘a’, ‘b’, ‘e’, and ‘f’, respectively) as well as to different possible local conformations of coils and turns (‘g’, ‘h’, ‘I’, and ‘j’) are much less numerous.

In order to account for the observed imbalance of different PB types, we trained two different models for PYTHIA predictions: one focused on maximizing the global accuracy of model predictions (global accuracy model) and one particularly efficient for the rare PB prediction (balanced accuracy model). The PYTHIA prediction results were then compared to LOCUSTRA methods in a ten-fold cross-validation as described in Section 4.5.

Both models outperform LOCUSTRA on average metrics. We improved the values of the MCC score by more than 10% for the global accuracy model (Table 1) and by more than 4% for the balanced accuracy model (Table 2). The global accuracy model provides the best average prediction performance compared to LOCUSTRA and performs especially well for the common PB classes such as ‘m’ (centra α-helix) and ‘d’ (central β-sheet). The balanced accuracy model was trained with weights anti-proportional to the PB class sizes and therefore allows us to obtain a more equilibrated distribution of performances among all PB classes. As a result, we obtain slightly lower accuracy in the prediction of overrepresented ‘m’ and ‘d’ PBs, while boosting the performance for the PBs corresponding to turns, helices, and sheet C-caps and N-caps. For instance, as compared to LOCUSTRA, we improve the true positive rate of prediction of class ‘j’ by 53% in the balanced accuracy model.

**Figure 2:**
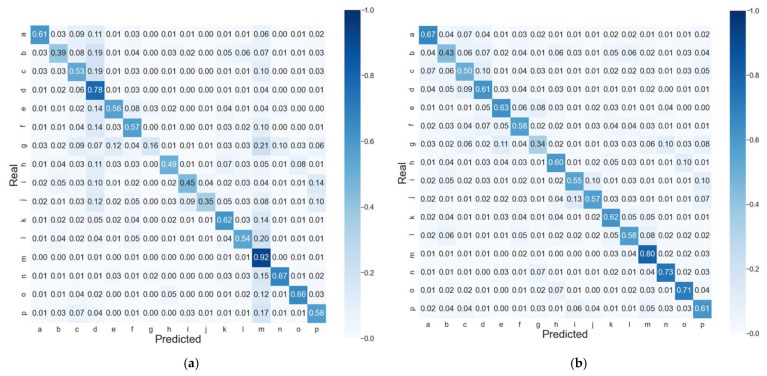
Normalized confusion matrices of PYTHIA predictions on the test dataset of best CV for the (**a**) global accuracy model and (**b**) balanced accuracy model.

The observed differences in the global accuracy and balanced accuracy models become even more obvious when considering the confusion matrices of predictions (Figure 2). While both models have quite pronounced diagonals corresponding to TPR, the mispredicted classes (off-diagonal elements) are distributed differently. In the balanced accuracy model (Figure 2b), the off-diagonal values are below 13% for all the classes and distributed randomly, while for the global accuracy model (Figure 2a), we observe an increased amount of protein regions misclassified as ‘m’ or ‘d’ classes. For instance, 21% of the very rare coil PB ‘g’ is predicted as in the middle of α-helix ‘m’ by the global accuracy model, while the balanced accuracy model makes this error only in 6% of cases. As a result, the TPR of ‘m’ and ‘d’ classes in the global accuracy model is higher than that of the balanced accuracy model by almost 17% and 11%, respectively, while in TPR, values are greater by at least 10% in the balanced accuracy model for all the coil PBs ‘g’, ‘h’, ‘I’, and ‘j’ (for the detailed differences between the global and balanced accuracy models, see Table S1 and Figure S1 for the LOCUSTRA confusion matrix).

**Figure 3:**
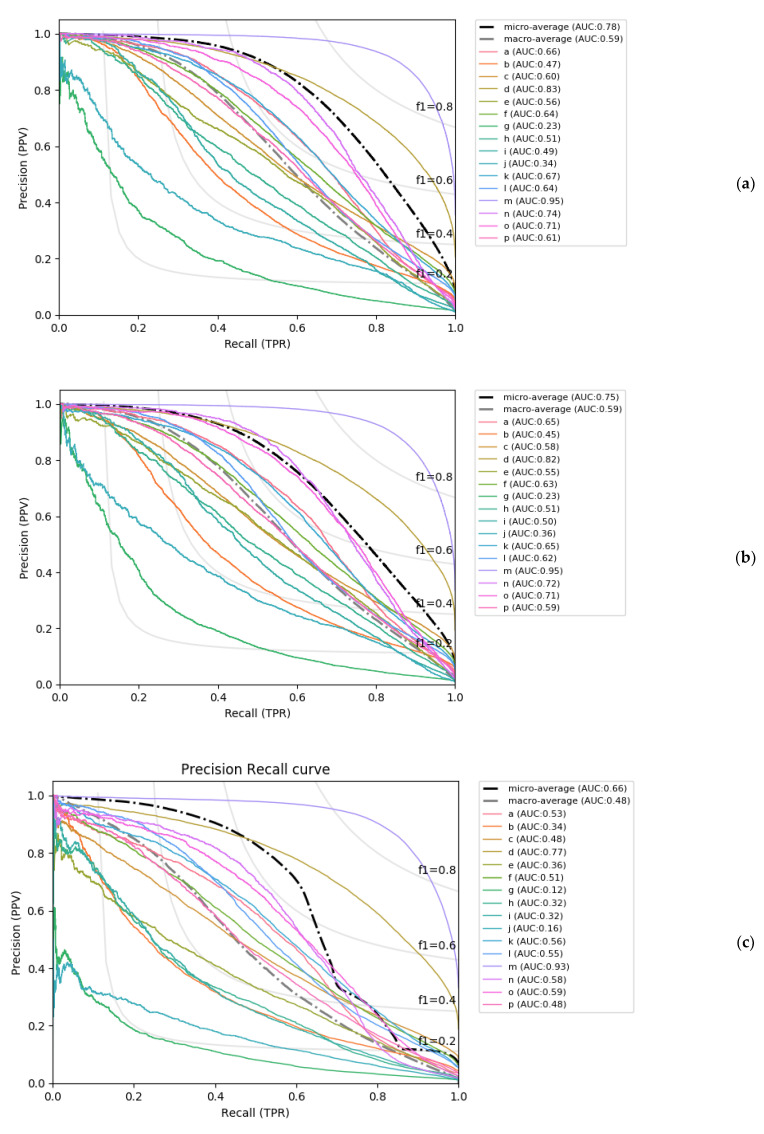
Precision-recall curves (PRC) of PYTHIA on the test dataset for (**a**) the best global accuracy model and (**b**) the best balanced accuracy model, and (**c**) the PRC curve of LOCUSTRA on the same test dataset.

Finally, it is important to note that PYTHIA’s greater performance as compared to LOCUSTRA derives from the much better prediction of the PB classes other than ‘m’ and ‘d’. Indeed, if we ignore these two classes and calculate the average TPR for the remaining 14 PBs, the obtained difference in the resulting Q_14_ becomes even more pronounced: the PYTHIA global and balanced accuracy models average 54% and 57.2% respectively, while LOCUSTRA Q_14_ equals only to 35.8%. These results further underline the important gain in prediction of the most complex PBs obtained by PYTHIA. In Figure 3, the precision-recall curves of PYTHIA and LOCUSTRA are also shown, which confirms the tendencies observed with others measures: very accurate prediction of classes ‘m’ and ‘d’, and low prediction accuracy for ‘g’ and ‘j’.

### 2.1. PYTHIA Performance on the CASP14 Free Modelling Targets

In order to estimate the performance of our models on an independent dataset, we have considered 10 targets from the CASP14 contest from the free modeling category. The selected targets were particularly challenging for 3D structure prediction considering they have particular folds and do not have any close homologs with resolved structures to be used as a template. Despite the complexity of the chosen structures, PYTHIA successfully predicted PB classes with an average accuracy of above 55% for both the global and balanced accuracy models (Table 3). Furthermore, the accuracy of PYTHIA predictions is greater than that of the LOCUSTRA predictions for all the targets. The overall improvement of the model accuracy varies from 2.3% to 21.7%.

**Figure 4:**
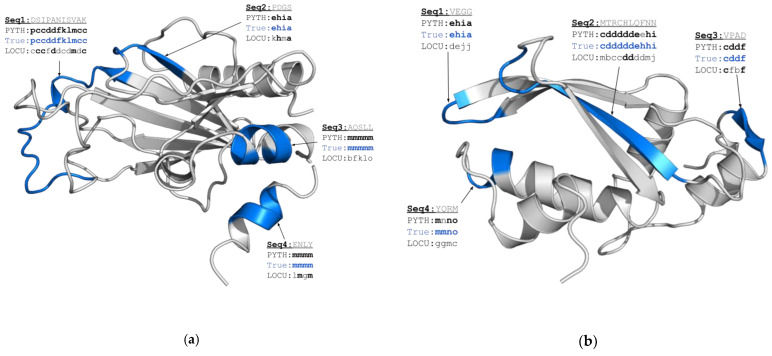
Comparison of the PB prediction results for two CASP14 targets: 7k7wA (**a**) and 6uf2A (**b**). In blue, we highlight protein regions in which we have observed the most pronounced difference in PYTHIA and LOCUSTRA predictions. The corresponding amino acid sequences are given in gray and the correctly predicted PBs are given in bold.

Among all the considered targets, PYTHIA provides a particularly impressive gain in prediction accuracy for the protein biofilm-related Se0862 protein from *Synechococcus elongatus* (6uf2A, 21.7%, Figure 4a) and for the N-terminal domain of Ssr4, which is a *Schizosaccharomyces pombe* chromatin-remodeling protein (7k7wA, 12.8%, Figure 4b). In Figure 4, we highlight the regions predicted correctly by PYTHIA and those misassigned by LOCUSTRA. PYTHIA performs better for both the ordered secondary structure regions and the coil regions of different sizes. Indeed, in contrast to LOCUSTRA, PYTHIA correctly identifies two α-helical fragments in Ssr4 (Seq3 and Seq4 in Figure 4a), as well as a long β-sheet (Seq2 in Figure 4b) and a short β-sheet motif in Se0862 (Seq3 in Figure 4b). Furthermore, PYTHIA better captures local conformations of the loops connecting secondary structure motifs. Indeed, for both considered examples, PYTHIA correctly assigned the transition between the two β-strands as “C-cap of β-sheet-coil-coil-N-cap of β-sheet” (Seq2 in Figure 4a and Seq1 in Figure 4b) and recognized a α-helix C-cap of α-helix transition (Seq4 in Figure 4b), while LOCUSTRA failed to attribute precise PB classes in these cases. Finally, PYTHIA also demonstrates a noticeable level of precision for the correct PB prediction for a long loop fragment of Ssr4 (Seq1 in Figure 4a).

### 2.2. Confidence of Predictions

**Figure 5:**
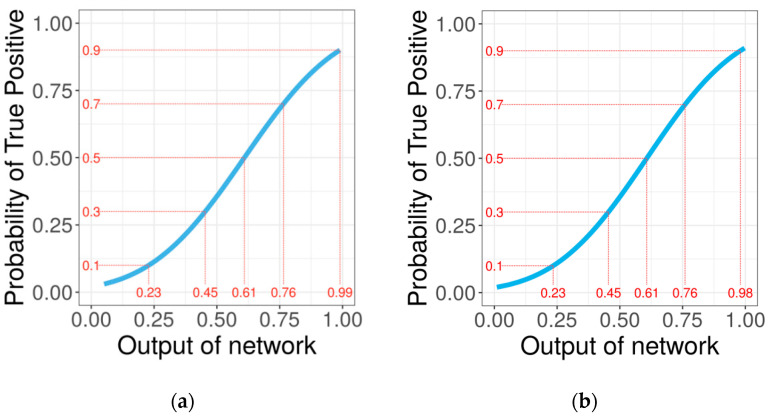
Fitting of the generalized linear models of the output probabilities of the balanced accuracy model (**a**) and global accuracy model (**b**) against the probability of true positives.

In addition to the predicted 1D PB profile, PYTHIA provides a more detailed output containing the network output probabilities for each PB at every sequence position. These probabilities can be related to the probability of true positives by a generalized logistic regression model (Figure 5). For network output probabilities below 0.5, the network is slightly over-confident: for example, when the model predicts a PB with a probability of 0.5, in reality, the probability that the PB is a true positive equal 0.4. However, globally and especially for probabilities higher than 0.5, PYTHIA output probabilities correlate quite well to the real TP probabilities.

## 3. Discussion

In the current study, we report a deep learning-based model for protein local conformation prediction in terms of Protein Blocks. PYTHIA demonstrates an important improvement of prediction performance over the reference SVM-based method LOCUSTRA. The observed gain derives from several factors, which include (i) the quantity of the available data on protein structures; (ii) the efficient protein sequence encoding; and (iii) the implementation and tuning of a deep learning model. In our method, each of these factors were chosen in accordance with the results reported for the similar problems of structural bioinformatics, such as secondary structure prediction [29,30,31,32] and flexibility prediction [8]. At the same time, the final network architecture as well as the combination of descriptors chosen for the sequence encoding are original and demonstrate the best results during model tuning.

The accuracy of PB prediction is limited by protein structural mobility as highly deformable regions (for example, located at protein loops) can adopt conformations corresponding to different PB types. As a result, the precision of PYTHIA predictions depends on the local variability of different protein positions. The expected value of this variability can be obtained through the analysis of the output probabilities of each PB class returned by PYTHIA. Indeed, the probability to obtain a true positive PB class for a position decreases with the increasing variability (entropy) of the returned network probabilities (Figure S2). The possible solution to increase the robustness of our predictions is to consider several PB classes predicted with the highest probabilities (top N) instead of the single most probable class. Indeed, for more than 93% of protein regions, the correct PB class is predicted as one of five most probable PB classes by the global accuracy model (Table S3, top 5). This value falls to 91% for the balanced accuracy model (Table S2, top 5). Therefore, a user can always access the more complete and reliable information on protein PB profiles by considering the detailed version of PYTHIA outputs.

Finally, we give the user the freedom to choose between the balanced accuracy and global accuracy models depending on the scientific problem. The first choice should be the global accuracy model considering it demonstrates a better overall performance. Nevertheless, in case of an expected or potentially important ratio of coiled regions, a user could prioritize using a balanced accuracy model in order to detect subtle variations in different coil conformations.

PYTHIA is available as a docker image, is easy to install, and runs on a PC, contrary to global methods such as AlphaFold 2 or RoseTTAFold. Our work points towards the multiple perspectives offered by PBs for the analysis of the dynamic properties of proteins and, more generally, conformers.

## 4. Materials and Methods

### 4.1. Dataset Preparation

The CulledPDB dataset was downloaded using the PISCES [33] server from Dunbrack lab (http://dunbrack.fccc.edu/PISCES.php accessed on 7 march 2019). The CulledPDB dataset was generated on 2019.03.07. The maximal sequence identity cutoff was set to 25%, the resolution cutoff was 2.2 Å, and the R-factor cutoff was 1.0. We downloaded the 11,047 chains (10544 unique PDBs IDs) of the CulledPDB list from the PDB [2]. We used a more recent version of the PDB than the one used by LOCUSTRA in their publication, which allows us to create much richer datasets and alignments as the number of protein structures more than tripled in 2020 compared to 2008 [34]. Nevertheless, we benchmarked PYTHIA with a cross-validation that guarantees the absence of redundancy between the training set and test data sets, and therefore provides an estimate of PYTHIA performance from below.

We built independent datasets for a 10-fold cross-validation. Each cross-validation is composed of a training (7954 PDB chains, 37 Go), validation (~1989 PDB chains, 9.6 Go) and test (1104 PDB chains, 4.4 Go) dataset.

### 4.2. Features Encoding

**Figure 6:**
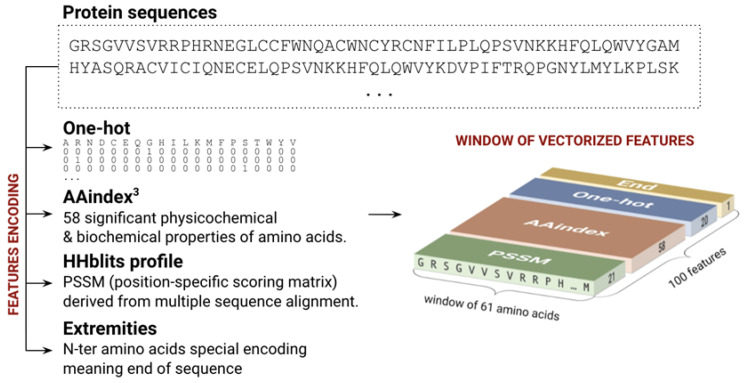
Sequence encoding used for prediction. We considered sequence fragments of 61 amino acids, each encoded by a vector of 100 features combining one-hot, AAindex, the PSSM profile, and the extremity flag.

Each amino acid is encoded by a vector of 100 features corresponding to a 20 one-hot encoding vector, 58 significant physicochemical properties called AAindexes [35], and the position specific scoring matrix (PSSM) [36,37] profile derived from a multiple sequence alignment (Figure 6). To obtain PSSM profiles, we performed a homologous search using HHblits [38] on the Uniclust database (version 09_2016) in three iterations with a 75% minimal coverage of the initial sequence, the E-value cut-off of 0.0001, and the maximal number of hits equal to 10,000. The multiple sequence alignments (MSA) were then filtered by HHfilter by minimal score per column equal to 30, by a target diversity of alignment equal to 20, and a maximal pairwise sequence identity of 99%. Finally, the resulting MSAs were translated into the PSSM profiles using a homemade utility implementing the pseudo-count algorithm of [36,37].

The LOCUSTRA generated profiles of the amino acid propensities for each sequence position were constructed with PSI-BLAST. Nonetheless, the new dataset we built for comparing the performances of both PYTHIA and LOCUSTRA was based on a more recent version of the UNIPROT database and on sequence profiles generated by HHBlits which was shown to be faster, considerably more sensitive, and produced alignments of much better quality than PSI-BLAST [19]. Therefore, the multiple sequence alignments we generate for LOCUSTRA predictions are more accurate, richer, and are likely to increase LOCUSTRA performance.

### 4.3. Deep Neural Network

**Figure 7:**
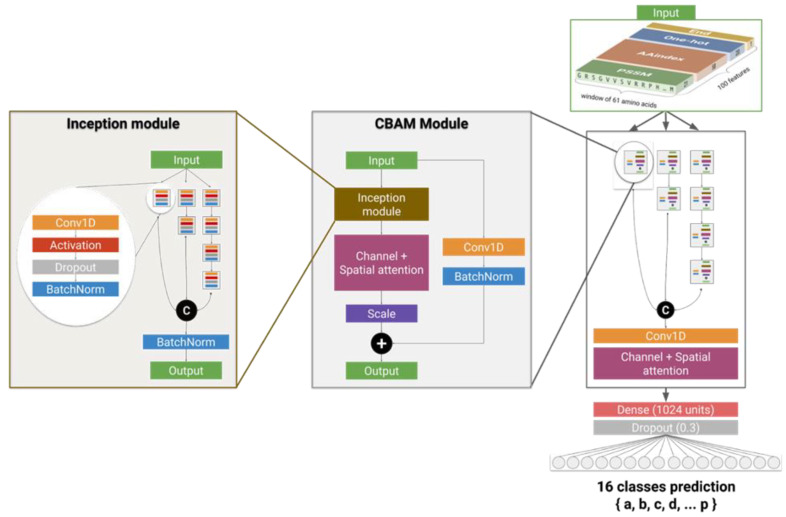
Deep neural network developed for the prediction of Protein Blocks. In the inception module, outputs of branches are concatenated (“C”). In the CBAM module, the residual connection is added to the main branch (“+”). The first 1D convolutional layer of the inception modules use a kernel size of 1 and the layers of higher depth levels use a kernel size of 3. The last 1D convolutional layer of the whole network uses a kernel of 11. All convolutional layers of the model have 128 filters.

The predictions were performed using a deep residual inception-inside-inception convolutional neural network (Figure 7). This network combines several state-of-the art neural network architectures and components that were shown to improve the performances of predictions in domains of computer vision as well as secondary structure prediction.

The network is based on two types of modules called inception [39] and convolutional block attention modules (CBAMs) [40] combined to a residual shortcut [39], together arranged into a global inception scheme. The inception module is composed of 7 subunits made of 4 simple layers: convolutional 1D, activation, dropout, and batch normalization. These units are interconnected to form a residual deep-inception scheme of 4 layers and 3 branches. Due to this inception scheme, these modules can extract features from input data at different scales of abstraction. The residual connections are used to reinject the original untransformed input data at each step of the transformation, which allows the network to not lose any important information during training. CBAMs improve the ability of the model to extract key features. CBAMs are composed of channel and spatial attention units through which the input data passes in order to obtain a reduced map of the most relevant features. Features are extracted in both channel and spatial-wise dimensions. The global network is composed of 7 CBAMs arranged in the same fashion as the inception module’s subunits, into 3 branches and 4 layers, in which each branch output is concatenated and the output is reduced through another channel and spatial attention module.

### 4.4. Network Training

#### 4.4.1. Hyperparameters

The network was developed in Python 3 with Tensorflow library (version 2.3.0), Pandas (v.1.1.5), Numpy (v1.18.5) and Tensorflow-addons (v0.13.0).

We used the RAdam optimizer [41] (a variant of the Adam optimizer whose adaptive learning rate is rectified) with a starting learning rate set to 5 × 10^−4^. After some semi-optimization of the hyperparameters, we figured that reducing the learning rate after half training gave better results; thus, at epoch 50, we reduced it to 5 × 10^−5^, then at epoch 60 to 2.5 × 10^−5^, and finally at epoch 65 to 1 × 10^−5^.

We used the Mish activation function [42]. The objective function used is categorical cross-entropy. We used a batch size of 256 and dropouts of 0.3. The model (16 017 386 parameters) was trained at 100 epochs during approximately 2 to 3 days on an NVIDIA Tesla V100 GPU (Nvidia Corporation, Santa Clara, CA, USA).

#### 4.4.2. Class Weighting

Our dataset is highly imbalanced mainly because of the over-representation of the ‘m’ and ‘d’ PBs. Therefore, we trained two models: with and without weighting. In order to balance the contribution of different PB types to the loss function, we applied class weights only during the training of the model. Class weighting adjusts weights of PB classes that are inversely proportional to the class frequencies in the dataset:

(1) $$W_{c}=\frac{n_{\mathrm{samples}}}{n_{classes}\times \;\mathrm{bincount}\left(y_{c}\right)},$$

where *c* is a PB class and *y_c_* is the array of the original class labels per sample.

### 4.5. Evaluation of Model Performances

We evaluate PYTHIA in a 10-fold cross-validation (CV). The whole dataset (11047 PDB chains) was randomly partitioned into 10 equal-sized sets. Each set is used as a test set (1104 PDB chains), while the rest is used as the training set (7954 PDB chains) from which 25% is used to create a validation set (1989 PDB chains) for evaluating the model at each epoch of the CV training.

Sensitivity/recall/true positive rate (TPR), F_1_-score, and Matthew’s correlation coefficient (MCC) were considered to evaluate the prediction performance of the model as follows:

(2) $$\mathrm{TPR}=\frac{\mathrm{TP}}{\mathrm{TP}\;+\;\mathrm{FN}},$$

(3) $$F_{1}=\frac{2\mathrm{TP}}{2\mathrm{TP}\;+\;\mathrm{FP}\;+\;\mathrm{FN}},$$

(4) $$\mathrm{MCC}=\frac{\mathrm{TP}\;\times \;\mathrm{TN}\;-\;\mathrm{FP}\;\times \;\mathrm{FN}}{\sqrt{\left(\mathrm{TP}\;+\;\mathrm{FP}\right)\left(\mathrm{TP}\;+\;\mathrm{FN}\right)\left(\mathrm{TN}\;+\;\mathrm{FP}\right)\left(\mathrm{TN}\;+\;\mathrm{FN}\right)}}\;,$$

where TP is the number of true positives, FP the number of false positives, TN the number of true negatives, and FN the number of false negatives. The statistical values of TPR and F_1_ vary between 0 and 1, and MCC varies between −1 and 1.

Finally, to estimate the expected variability of the predicted PBs at a given protein position, we used the following formula of informational entropy N*_eq_*:

(5) $$N_{eq}=\;\exp\left(\overset{16}{\underset{1}{\sum }}p_{i}\ln(p_{i})\right)$$

where *p*_i_ is the network output probability for PB class *i*.

## Figures and Tables

**Table 1:** Evaluation of performances of the global accuracy PYTHIA model compared to the reference method LOCUSTRA. Several statistical measures were used: True Positive Rate (TPR), F1-score and Matthew Correlation Coefficient (MCC). The DIFF columns represent in green the gain of performances in favor of PYTHIA and in red in favor of LOCUSTRA. The width of bars is scaled ac-cording to the highest value of the corresponding metric. We present two metrics for average: “Macro” average (averaging the unweighted mean per PB class) and “Micro” average (averaging the total true positives, false negatives and false positives). Micro average of TPR corresponds to Q_16_.

	TPR/SENS		F1 Score				MCC		
PB	LOCUSTRA	PYTHIA	DIFF	LOCUSTRA	PYTHIA	DIFF	LOCUSTRA	PYTHIA	DIFF
a	47.6	**60.7**	+13.1	53.9	**62.9**	+9.0	52.9	**61.2**	+8.3
b	20.6	**39.2**	+18.6	29.2	**45.1**	+16.0	30.2	**42.9**	+12.7
c	37.1	**52.8**	+15.7	44.6	**55.5**	+10.9	41.6	**50.6**	+9.0
d	69.0	**78.4**	+9.4	69.9	**75.4**	+5.5	63.0	**67.7**	+4.7
e	28.1	**55.9**	+27.8	35.9	**55.7**	+19.9	36.3	**54.4**	+18.1
f	37.6	**57.4**	+19.8	46.7	**60.2**	+13.5	45.5	**56.8**	+11.3
g	3.4	**16.0**	+12.6	6.2	**22.9**	+16.7	10.6	**24.8**	+14.2
h	24.6	**49.4**	+24.8	33.1	**51.6**	+18.5	34.4	**50.3**	+15.9
i	26.4	**45.0**	+18.6	34.6	**49.3**	+14.7	35.7	**48.6**	+12.9
j	2.9	**35.2**	+32.3	5.4	**37.2**	+31.8	10.1	**36.6**	+26.5
k	43.5	**61.7**	+18.2	52.7	**63.0**	+10.3	52.0	**60.3**	+8.3
l	38.3	**54.1**	+15.8	50.0	**59.0**	+9.0	50.8	**56.6**	+5.8
m	93.0	91.8	−1.2	71.1	**87.2**	+16.2	56.0	**77.7**	+21.7
n	51.0	**67.2**	+16.2	58.8	**67.1**	+8.3	58.9	**66.3**	+7.4
o	50.6	**65.8**	+15.2	56.6	**65.7**	+9.0	56.1	**64.5**	+8.4
p	37.1	**57.6**	+20.5	46.0	**57.1**	+11.1	46.0	**55.1**	+9.1
**Macro**	38.2	**55.5**	+17.3	43.4	**57.2**	+13.8	42.5	**54.7**	+12.2
**Micro**	60.8	**71.1**	+10.3	60.8	**71.1**	+10.3	58.2	**68.5**	+10.3

The best performances obtained for each target are in bold.

**Table 2:** Evaluation of the performances of the balanced accuracy PYTHIA model compared to the LOCUSTRA reference method. Several statistical measures were used: true positive rate (TPR), F1-score, and Matthew′s correlation coefficient (MCC). The DIFF columns in green represent the gain of performances in favor of PYTHIA and in red, in favor of LOCUSTRA. The width of bars is scaled according to the highest value of the corresponding metric. We present two metrics for average: “macro” average (averaging the unweighted mean per class) and “micro” average (averaging the total of the true positives, false negatives, and false positives). The micro average of TPR corresponds to Q_16_.

	TPR/SENS			F1 Score			MCC		
PB	LOCUSTRA	PYTHIAb	DIFF	LOCUSTRA	PYTHIAb	DIFF	LOCUSTRA	PYTHIAb	DIFF
a	47.6	**67.0**	+19.4	53.9	**58.5**	+4.6	52.9	**56.4**	+3.5
b	20.6	**43.0**	+22.4	29.2	**41.4**	+12.2	30.2	**37.4**	+7.2
c	37.1	**49.9**	+12.8	44.6	**52.6**	+8.0	41.6	**46.9**	+5.3
d	69.0	61.5	−7.5	69.9	**70.4**	+0.5	63.0	**63.4**	+0.4
e	28.1	**62.7**	+34.6	35.9	**51.6**	+15.7	36.3	**50.6**	+14.3
f	37.6	**57.4**	+19.8	46.7	**57.0**	+10.3	45.5	**52.6**	+7.1
g	3.4	**34.1**	+30.7	6.2	**22.7**	+16.5	10.6	**22.4**	+11.8
h	24.6	**59.7**	+35.1	33.1	**48.0**	+15.0	34.4	**47.1**	+12.7
i	26.4	**55.3**	+28.9	34.6	**46.2**	+11.7	35.7	**45.5**	+9.8
j	2.9	**56.5**	+53.6	5.4	**35.9**	+30.5	10.1	**37.5**	+27.4
k	43.5	**61.7**	+18.2	52.7	**59.4**	+6.7	52.0	**56.0**	+4.0
l	38.3	**57.9**	+19.6	50.0	**54.2**	+4.2	50.8	**50.5**	−0.3
m	93.0	80.3	−12.7	71.1	**85.9**	+14.9	56.0	**76.7**	+20.7
n	51.0	**72.8**	+21.8	58.8	**60.3**	+1.6	58.9	**60.1**	+1.2
o	50.6	**71.2**	+20.6	56.6	**59.8**	+3.2	56.1	**59.0**	+2.8
p	37.1	**60.5**	+23.4	46.0	**51.4**	+5.5	46.0	**49.2**	+3.2
**Macro**	38.2	**59.5**	+21.3	43.4	**53.5**	+10.1	42.5	**50.7**	+8.2
**Micro**	60.8	**65.4**	+4.6	60.8	**65.4**	+4.6	58.2	**62.0**	+3.8

The best performances obtained for each target are in bold.

**Table 3:** Q_16_ on the CASP14 free modelling targets (%) for two PYTHIA models (the balanced and global accuracy) and LOCUSTRA reference PB prediction method.

CASP 14Targets	Length	PYTHIA		LOCUSTRA
		Balanced	Global	
6uf2A	125	**69.4**	**69.4**	47.7
6xc0C	105	**63.9**	61.1	59.5
6y4fA	141	56.9	**57.7**	48.2
6ya2A	199	43.5	**47.8**	45.5
6zycA	148	66.0	**68.1**	57.0
7d2oA	174	36.6	**42.1**	30.4
7jtlA	107	27.7	**43.6**	37.6
7k7wA	590	66.9	**69.2**	56.4
7m7aA	197	59.1	**69.6**	64.5
7m7aB	590	69.1	**70.5**	66.5
**Mean**		**55.9**	**59.9**	51.3

The best performances obtained for each target are in bold.

## Data Availability

The code and data are available online in the Github repository https://github.com/DSIMB/PYTHIA.

## Supplementary Materials

The following are available online at https://www.mdpi.com/article/10.3390/ijms22168831/s1.
