# Supplementary material for: PYTHIA: Deep Learning Approach for Local Protein Conformation Prediction
Source: Int J Mol Sci. 2021 Aug 17;22(16):8831. doi: 10.3390/ijms22168831 (PMC8396346; doi:10.3390/ijms22168831)
Supplement: Supplementary file 1 [file ijms-22-08831-s001.zip › ijms-1309701-supplementary 2.pdf]

International Journal of
Molecular Sciences

MDPI

Supplementary materials

Table S1: Comparison of PYTHIA global accuracy model against PYTHIA balanced accuracy model.

	TPR/SENS			F1 Score			MCC		
PB	PYTH	PYTHb	DIFF	PYTH	PYTHb	DIFF	PYTH	PYTHb	DIFF
**a**	60.7	67.0	6.3	62.9	58.5	4.5	61.2	56.4	4.8
**b**	39.2	43.0	3.8	45.1	41.4	3.8	42.9	37.4	5.5
**c**	52.8	49.9	2.9	55.5	52.6	2.9	50.6	46.9	3.7
**d**	78.4	61.5	16.9	75.4	70.4	5.0	67.7	63.4	4.3
**e**	55.9	62.7	6.7	55.7	51.6	4.1	54.4	50.6	3.8
**f**	57.4	57.4	0.0	60.2	57.0	3.2	56.8	52.6	4.2
**g**	16.0	34.1	18.0	22.9	22.7	0.2	24.8	22.4	2.4
**h**	49.4	59.7	10.3	51.6	48.0	3.5	50.3	47.1	3.3
**i**	45.0	55.3	10.3	49.3	46.2	3.1	48.6	45.5	3.1
**j**	35.2	56.5	21.3	37.2	35.9	1.3	36.6	37.5	0.9
**k**	61.7	61.7	0.1	63.0	59.4	3.6	60.3	56.0	4.3
**l**	54.1	57.9	3.8	59.0	54.2	4.8	56.6	50.5	6.0
**m**	91.8	80.3	11.5	87.2	85.9	1.3	77.7	76.7	1.0
**n**	67.2	72.8	5.6	67.1	60.3	6.7	66.3	60.1	6.2
**o**	65.8	71.2	5.4	65.7	59.8	5.8	64.5	59.0	5.6
**p**	57.6	60.5	2.9	57.1	51.4	5.7	55.1	49.2	5.9
**Macro**	55.5	59.5	4.0	57.2	53.5	3.7	54.7	50.7	4.0
**Micro**	71.1	65.4	5.7	71.1	65.4	5.7	68.5	62.0	6.5

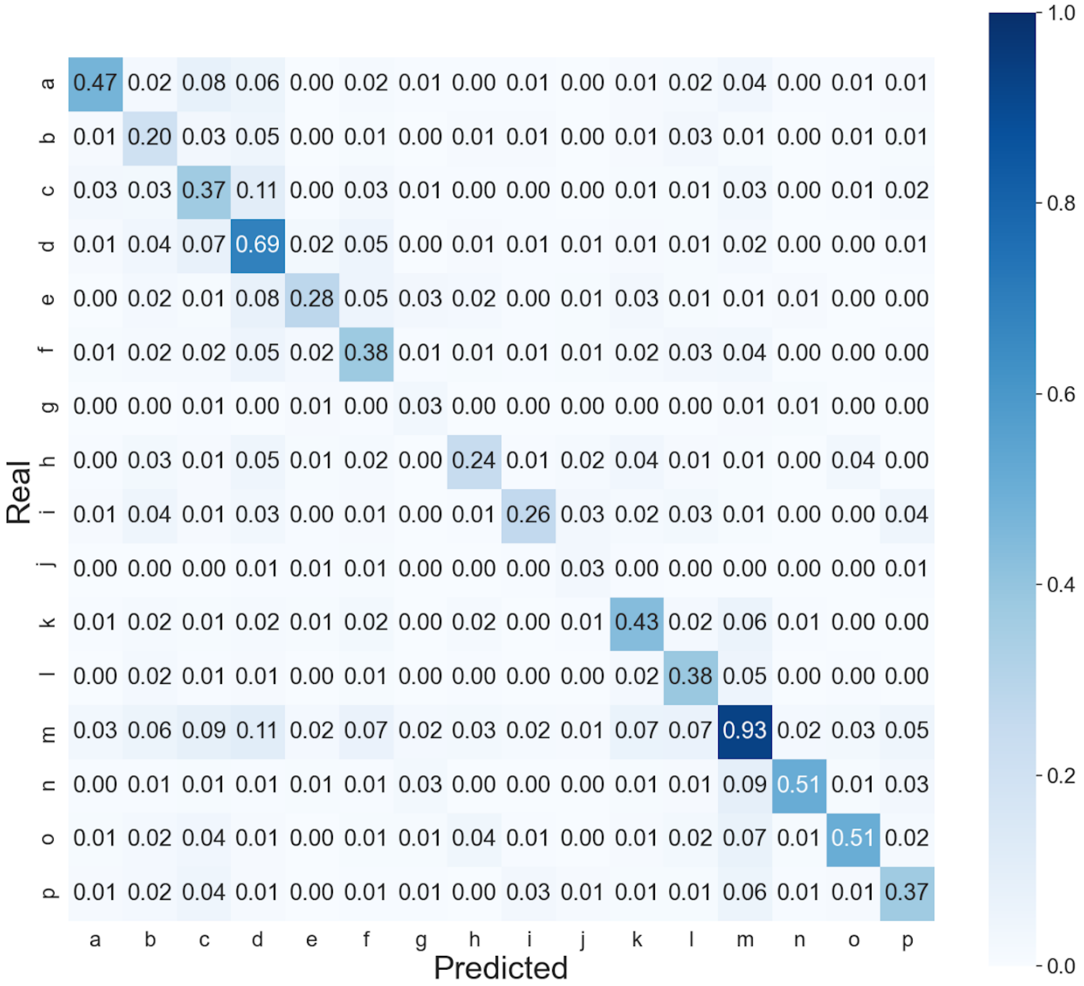

Figure S1: Normalized confusion matrix of predictions of LOCUSTRA on the test dataset.

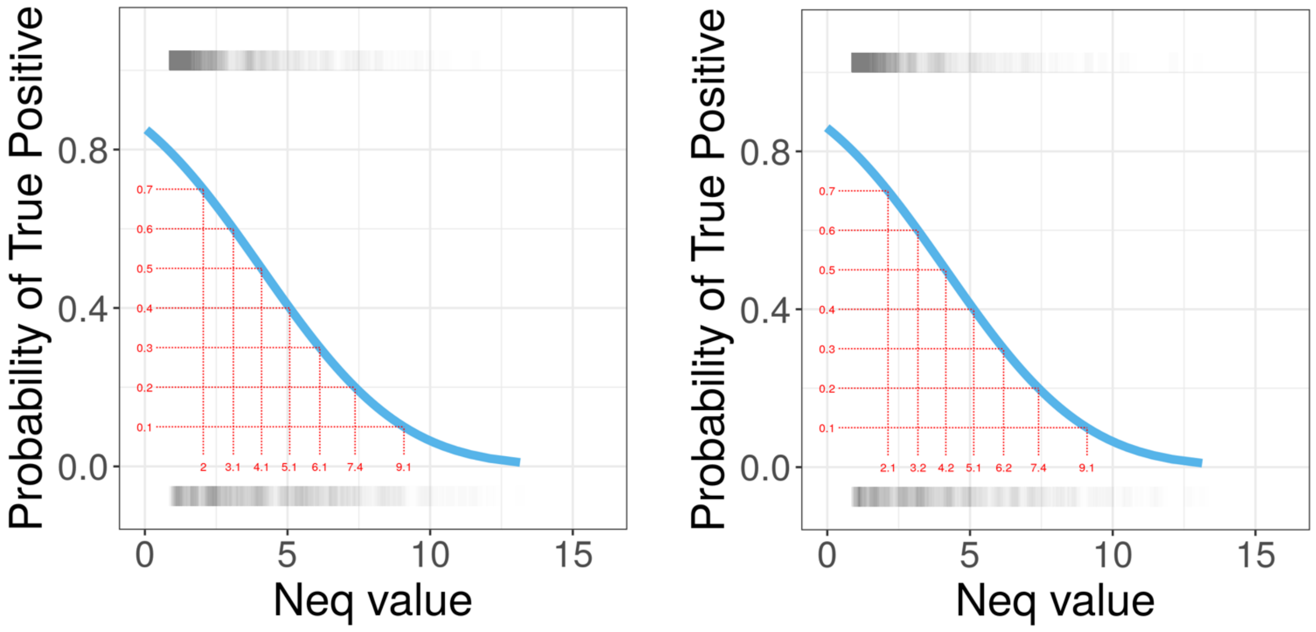

(a) (b)

Figure S2: Probability of true positive as a function of the local variability of PB for global accuracy mode (a) and balanced accuracy model (b). N_{eq} quantifies the average number of PBs at a given position in the protein sequence from 1 to 16 (fully random distribution of PBs). This value is an entropy based index (see Materials and Methods formula 5). The curve is obtained by fitting a generalized linear regression model.

Table S2. True positive rates for the balanced accuracy PYTHIA model.

PBs	TOP 1	TOP 2	TOP 3	TOP 4	TOP 5
**a**	66.96 ± 1.22	74.1 ± 1.28	80.67 ± 1.18	84.74 ± 1.09	87.96 ± 0.94
**b**	43.03 ± 2.6	63.62 ± 2.76	77.11 ± 2.49	86.28 ± 2.11	91.08 ± 1.44
**c**	49.89 ± 1.34	72.05 ± 1.47	81.24 ± 1.44	87.29 ± 1.15	91.84 ± 0.94
**d**	61.48 ± 1.87	76.51 ± 1.73	84.4 ± 1.52	88.52 ± 1.38	91.48 ± 1.15
**e**	62.66 ± 1.81	75.63 ± 1.62	82.09 ± 1.32	85.12 ± 1.2	87.51 ± 1.06
**f**	57.38 ± 2.08	72.12 ± 1.75	80.89 ± 1.32	87.42 ± 0.8	91.31 ± 0.74
**g**	34.05 ± 1.44	56.92 ± 2.7	66.86 ± 2.93	74.1 ± 2.85	79.21 ± 2.5
**h**	59.66 ± 1.68	74.24 ± 1.27	79.33 ± 1.26	83.06 ± 1.03	86.19 ± 0.86
**i**	55.33 ± 1.79	69.61 ± 1.73	75.97 ± 1.54	79.14 ± 1.42	81.9 ± 1.38
**j**	56.53 ± 3.44	69.18 ± 2.56	76.29 ± 2.27	79.68 ± 2.09	82.24 ± 1.83
**k**	61.73 ± 1.42	73.14 ± 1.46	81.56 ± 1.35	86.6 ± 1.12	90.44 ± 0.89
**l**	57.91 ± 1.76	75.06 ± 1.32	82.02 ± 1.14	86.3 ± 0.97	89.89 ± 0.89
**m**	80.34 ± 1.46	87.91 ± 1.03	91.53 ± 0.88	93.7 ± 0.75	94.97 ± 0.66

**n**	72.82 ± 2.28	81.43 ± 1.73	86.26 ± 1.38	88.5 ± 1.18	90.16 ± 1.18
**o**	71.19 ± 1.86	80.51 ± 1.03	84.15 ± 0.89	86.93 ± 0.85	89.65 ± 0.8
**p**	60.46 ± 2.16	75.23 ± 1.83	82.94 ± 1.21	87.28 ± 0.94	90.65 ± 0.65
**Micro**	65.56 ± 1.06	78.36 ± 0.88	84.95 ± 0.75	88.96 ± 0.64	91.73 ± 0.51
**Macro**	59.46 ± 1.23	73.57 ± 0.89	80.84 ± 0.73	85.3 ± 0.62	88.54 ± 0.48

Table S3. True positive rates for the global accuracy PYTHIA model. "Macro" average (averaging the unweighted mean per class) and "Micro" average (averaging the total true positives, false negatives and false positives).

PBs	TOP 1	TOP 2	TOP 3	TOP 4	TOP 5
**a**	60,65 ± 1,55	68,32 ± 1,5	77,32 ± 1,13	82,07 ± 0,88	85,48 ± 0,71
**b**	39,23 ± 2,12	58,5 ± 2,18	72,62 ± 1,85	82,8 ± 1,36	88,73 ± 1,03
**c**	52,82 ± 1,4	77,85 ± 1,09	87,37 ± 0,66	92,07 ± 0,45	95,42 ± 0,28
**d**	78,38 ± 1,2	89,1 ± 0,9	93 ± 0,67	95,12 ± 0,45	96,55 ± 0,33
**e**	55,93 ± 1,02	70,22 ± 1,36	78,03 ± 1,02	82,13 ± 1,09	85,47 ± 0,98
**f**	57,42 ± 1,33	74,05 ± 0,97	82,87 ± 0,74	89,07 ± 0,6	92,3 ± 0,54
**g**	16,02 ± 0,74	34,25 ± 1,87	49,02 ± 2,31	62,05 ± 2,56	70,27 ± 1,68
**h**	49,37 ± 1,91	64,07 ± 1,9	71,48 ± 1,66	76,88 ± 1,67	81,32 ± 1,26
**i**	45,03 ± 1,66	58,9 ± 1,57	66,68 ± 2,09	72,25 ± 1,7	76,4 ± 1,83
**j**	35,23 ± 2,66	55,73 ± 2,05	68,97 ± 2,22	76,97 ± 2,31	81,7 ± 1,77
**k**	61,65 ± 1,47	73,87 ± 1,39	82,5 ± 1,25	87,2 ± 1,04	90,82 ± 0,74
**l**	54,13 ± 1,13	74,53 ± 1,07	82,27 ± 0,82	86,27 ± 0,7	89,72 ± 0,5
**m**	91,82 ± 0,38	96,37 ± 0,23	97,6 ± 0,18	98,23 ± 0,15	98,7 ± 0,13
**n**	67,17 ± 2,02	78,57 ± 1,49	83,87 ± 1,13	86,47 ± 1,11	88,3 ± 1,29
**o**	65,77 ± 1,8	75,87 ± 1,76	80,15 ± 1,85	83,52 ± 1,62	86,53 ± 1,41
**p**	57,55 ± 1,87	72,27 ± 1,35	79,65 ± 1,13	84,98 ± 1	88,9 ± 0,55
**Micro**	71,13 ± 0,94	82,72 ± 0,8	88,15 ± 0,59	91,4 ± 0,45	93,65 ± 0,33
**Macro**	55,5 ± 1,31	70,17 ± 1,16	78,35 ± 1	83,62 ± 0,89	87,28 ± 0,7
